# Comparing Entrustable Professional Activity Scores Given by Faculty Physicians and Senior Trainees to First-Year Residents

**DOI:** 10.7759/cureus.25798

**Published:** 2022-06-09

**Authors:** Steven J Katz, Dennis Wang

**Affiliations:** 1 Medicine, University of Alberta, Edmonton, CAN

**Keywords:** work-based assessment, competency-based medical education, entrustable pofessional activities, pgme postgraduate medical education, postgraduate medical education

## Abstract

Introduction

Competency by Design (CBD) began on July 1, 2019, for postgraduate year 1 (PGY1) Canadian Core Internal Medicine (CIM) residents. Many entrustable professional activity (EPA) observations allow for assessment by either a faculty physician, senior medicine resident (SMR), or subspecialty resident (SSR). However, few studies exist that compare EPA scores and comments given by faculty vs senior trainees (SMRs and SSRs). This study aimed to identify differences in EPA scores and comments given to PGY1 residents by faculty physicians vs senior trainees.

Methods

Scores and comments of EPAs completed between July 1, 2019, and June 30, 2020, for 35 CIM PGY1 residents were extracted anonymously from the University of Alberta CBD platform. Scores from faculty vs senior trainees were compared with the Mann-Whitney U test and the Kruskal-Wallis test. Word counts for positive and constructive comments written by faculty vs senior trainees were compared with the independent t-test and one-way ANOVA. The most common two-word phrases in comments were identified with QI Macros software (Denver, CO: KnowWare International, Inc.).

Results

A total of 2226 EPAs were observed. Faculty physicians gave significantly lower EPA scores overall compared to senior trainees (U = 501706, P <0.001). Constructive comments written by faculty (M = 14.06, SD = 16.84) had lower word counts compared to senior trainees (M = 15.85, SD = 16.43) for overall EPAs (t{2224} = -2.528, P = 0.012).

Conclusion

Faculty physicians gave lower EPA scores and had lower word counts on constructive comments, compared to senior trainees. These results may help the ongoing implementation of Competence by Design.

## Introduction

With the increased attention to competency-based medical education (CBME) over the past few years, the Royal College of Physicians and Surgeons of Canada (RCPSC) recently implemented its version of CBME called Competence by Design (CBD), first formally launching in July 2017 with Anesthesiology and Otolaryngology - Head and Neck Surgery, and Core Internal Medicine (CIM) formally adopting CBD later in July 2019 [1-3]. Faculty physicians, senior medicine residents (SMRs), and subspecialty residents (SSRs - a resident who has completed their Core Internal Medicine training {postgraduate years 1-3 "PGY1-3"} and are now completing subspecialty training {PGY4-6}) assess junior residents in performing entrustable professional activities (EPAs), which are the essential tasks of the specialty the resident is training in [4-6]. With SMRs and SSRs becoming more involved in assessing their junior colleagues with EPAs, it is possible there are differences that exist between assessments from senior trainees (SMRs and SSRs) and faculty physicians, and if so, it is possible these differences have consequences on junior resident assessment. 

Individual EPA observations are scored on a 1-5 entrustment scale adapted from the Ottawa Clinic Assessment Tool; an EPA score of 1 equates to “I had to do”, meaning the supervisor had to completely take over the task, and a score of 5 equates to “I didn’t need to be there”[7], meaning the learner was able to perform the task competently and safely without the theoretical presence of a supervisor. The assessor can also write positive and constructive comments. These EPA assessments form the basis of resident progression through the four stages of CBD: transition to discipline (TD), foundations of discipline (FD), core of discipline, and transition to practice [6,8].

As outlined by conceptual models from Kogan et al. and Berendonk et al., the cognitive process of assessing trainees is complex and prone to influence by multiple factors, including the assessor’s characteristics, their frame of reference for standard performance, and the context of the clinical encounter [9,10]. These factors likely influence senior trainees. Additionally, previous studies have looked at differences between faculty and trainee assessors for other modes of assessment, such as objective structured clinical examinations (OSCEs) and workplace-based assessments. There is conflicting evidence about which group is more lenient; some studies show that trainees give higher scores on such assessments than faculty [11-13] and other studies show that trainees score lower than faculty [14,15]. However, at this time, there are few studies that compare differences in EPA scores or comments given by faculty physicians, SMRs, and SSRs. If one group is more lenient than another, this could have unintended consequences for learners and programs. For example, is it possible that learners will seek out assessment from a more lenient group? Would this information influence which supervisors are allowed to provide assessment? Does this information influence how programs interpret assessment data?

This study compares the scores and comments for TD and FD EPAs given by faculty physicians vs senior trainees to PGY1 residents in the CIM residency program at the University of Alberta.

## Materials and methods

EPA scores

Transition to discipline and foundations of discipline EPA scores and comments for 35 University of Alberta CIM PGY1s from July 2019 to June 2020 were extracted from CBME.med, the local CBD electronic platform. Identifying information, such as the names of PGY1 residents, faculty physicians, SMRs, and SSRs were removed. Only clinical work-based observations were included; observations done during simulations or OSCEs were excluded. There were three TD EPAs (TD1, TD2, TD3) and seven FD EPAs (FD1 to FD7) [8]. EPA scores given by faculty physicians vs senior trainees were compared with the Mann-Whitney U test. EPA scores given by faculty physicians vs SMRs vs SSRs were compared with the Kruskal-Wallis H test, with post-hoc pairwise comparisons done by the Mann-Whitney U test. For EPAs FD5 and FD6, SMRs and SSRs were grouped together in CBME.med as one choice when selecting the type of observer; thus, the EPA scores given by faculty physicians vs SMRs vs SSRs could only be compared for EPAs TD1 to FD4b.

EPA comments: word counts and most common phrases

Word counts for positive and constructive comments written by faculty physicians vs senior trainees were compared with the independent t-test. Word counts for both positive and constructive EPA comments written by faculty physicians vs SMRs vs SSRs were compared with one-way ANOVA, with post-hoc testing done with Tukey's honestly significant difference test (Tukey's HSD). For EPAs FD5 and FD6, SMRs and SSRs were grouped as one choice when selecting the type of observer; thus, EPA scores between faculty physicians vs SMRs vs SSRs could only be compared for EPAs TD1 to FD4b.

QI Macros software (Denver, CO: KnowWare International, Inc.) was used to find the top ten most common two-word phrases for both positive and constructive EPA comments provided by faculty physicians and senior trainees. The University of Alberta Medical Ethics Board approved this project (#Pro00097054). The research was conducted in accordance with the Declaration of Helsinki.

## Results

EPA scores: general statistics

A total of 2226 EPAs completed by 35 PGY1 CIM residents were observed for TD1-FD6. Faculty physicians observed 1174 EPAs, with a mean score of 4.56 ± 0.639. Senior trainees observed 1052 EPAs, with a mean score of 4.80 ± 0.423. Out of the total 2226 EPAs observed, 1909 EPAs were observed for TD1-FD4a. Faculty physicians observed 989 EPAs, with a mean score of 4.54 ± 0.652. SMRs observed 496 EPAs, with a mean score of 4.85 ± 0.38. SSRs observed 424 EPAs, with a mean score of 4.73 ± 0.48 (Table 1).

**Table 1:** EPA scores, organized by stage, given by faculty physicians, SMR, and SSR EPA: entrustable professional activity; SMR: senior medicine resident; SSR: subspecialty resident; TD: transition to discipline; FD: foundations of discipline

	N	Mean	SD	Median	Q1	Q3	IQR	Min	Max	Range
Overall (TD1-FD6)										
Faculty	1174	4.56	0.639	5	4	5	1	2	5	3
SMR/SSR	1052	4.8	0.423	5	5	5	0	3	5	2
Overall (TD1-FD4b)										
Faculty	989	4.54	0.652	5	4	5	1	2	5	3
SMR	496	4.85	0.38	5	5	5	0	3	5	2
SSR	424	4.73	0.48	5	4	5	1	3	5	2
TD (TD1-3)										
Faculty	199	4.31	0.746	4	4	5	1	2	5	3
SMR/SSR	190	4.66	0.508	5	4	5	1	3	5	2
SMR	94	4.78	0.419	5	5	5	0	4	5	1
SSR	96	4.54	0.56	5	4	5	1	3	5	2
FD (FD1-6)										
Faculty	975	4.62	0.602	5	4	5	1	2	5	3
SMR/SSR	862	4.84	0.394	5	5	5	0	3	5	2
FD (FD1-4b)										
Faculty	790	4.6	0.612	5	4	5	1	2	5	3
SMR	402	4.87	0.369	5	5	5	0	3	5	2
SSR	328	4.79	0.439	5	5	5	0	3	5	2

EPA scores: faculty physicians vs senior trainees

Mann-Whitney U test showed that EPA scores given by faculty physicians were significantly lower than EPA scores given by senior trainees when comparing EPA scores from TD1-FD6, (U = 501706, P <0.001), TD1-3 (U = 14164, P <0.001), and FD1-6 (U = 344971, P <0.001) (Table 2). When looking at each EPA individually, the Mann-Whitney U test showed that faculty physicians gave lower scores compared to senior trainees for all EPAs except for FD4a and FD4b, in which there was no significant difference. No SMRs or SSRs gave EPA scores for FD3, as this EPA can only be observed by faculty physicians.

**Table 2:** Mann-Whitney U test comparison of EPA scores between faculty physicians and senior trainees. EPA: entrustable professional activity; TD: transition to discipline; FD: foundations of discipline

	Mean rank - faculty	Mean rank - senior trainee	U	P-value (2-tailed)
Overall (TD1-FD6)	1014.85	1223.59	501,706	<0.001
TD (TD1-3)	171.18	219.95	14,164	<0.001
FD (FD1-6)	841.82	1006.3	344,971	<0.001

EPA scores: faculty physicians vs SMRs vs SSRs

The Kruskal-Wallis H test showed that there was a significant difference in TD1-FD4a EPA scores given by faculty physicians vs SMRs vs SSRs (H = 100.091, P <0.001). Pairwise comparisons done by the Mann-Whitney U test showed that faculty physicians gave lower scores compared to SMRs (U = 185310, P <0.001) and SSRs (U = 180459, P <0.001). SSRs gave lower scores compared to SMRs (U = 93633.5, P <0.001) (Table 3).

**Table 3:** Comparison of EPA scores between faculty physicians, SMR vs SSR EPA: entrustable professional activity; SMR: senior medicine resident; SSR: subspecialty resident; TD: transition to discipline; FD: foundations of discipline

	Kruskal-Wallis H test			Mean rank - faculty	Mean rank - SMR	Mean rank - SSR	Mann-Whitney U test	
	H	df	P-value				U	P-value (2-tailed)
Overall (TD1-FD4)								
Faculty vs SMR vs SSR	100.091	2	<0.001	864.84	1099.11	996.72	-	-
Faculty vs SMR	-	-	-	682.37	863.89	-	185,310	<0.001
Faculty vs SSR	-	-	-	677.47	-	775.89	180,459	<0.001
Faculty vs SMR	-	-	-	-	483.72	433.33	93633.5	<0.001
TD (TD1-3)								
Faculty vs SMR vs SSR	31.125	2	<0.001	171.18	239.79	200.53	-	-
Faculty vs SMR	-	-	-	130.62	181.68	-	6093.5	<0.001
Faculty vs SSR	-	-	-	140.56	-	163.43	8070.5	0.017
SSR vs SMR	-	-	-	-	105.61	85.6	3561.5	0.002
FD (FD1-4b)								
Faculty vs SMR vs SSR	73.905	2	<0.001	692.68	859.51	802.5	-	-
Faculty vs SMR	-	-	-	552.49	682.99	-	124020	<0.001
Faculty vs SSR	-	-	-	535.69	-	616.84	110751	<0.001
SSR vs SMR	-	-	-	-	378.02	350.15	60894	0.005

The Kruskal-Wallis H test showed that there was a significant difference in TD1-3 EPA scores given by faculty physicians vs SMRs vs SSRs (H = 31.125, P <0.001). Pairwise comparisons done by the Mann-Whitney U test showed that faculty physicians gave lower scores compared to SMRs (U = 6093.5, P <0.001) and SSRs (U = 8070.5, P =0.017). SSRs gave lower scores compared to SMRs (U = 3561.5, P <0.002) (Table 3).

The Kruskal-Wallis H test showed that there was a significant difference in FD1-4b EPA scores given by faculty physicians vs SMRs vs SSRs (H = 73.905, P <0.001). Pairwise comparisons done by the Mann-Whitney U test showed that faculty physicians gave lower scores compared to SMRs (U = 124020, P <0.001) and SSRs (U = 110751, P <0.001). SSRs gave lower scores compared to SMRs (U = 60894, P =0.005) (Table 3).

When looking at each EPA individually, the Kruskal-Wallis H test and pairwise comparisons with the Mann-Whitney U test showed that faculty physicians gave lower scores compared to SMRs for all EPAs except for TD3, FD4a, and FD4b, in which there was no significant difference. Faculty physicians gave lower scores than SSRs for EPAs FD1, FD2a, and FD2b; there was no significant difference between faculty physicians and SSRs for the other EPAs. SSRs gave lower EPA scores than SMRs for EPAs TD1 and TD2; there was no significant difference between SSRs and SMRs for the other EPAs.

EPA comments: comparing word counts

For positive EPA comments, there was no significant difference in word counts between faculty physicians (M = 19.15, SD = 20.16) and senior trainees (M = 18.73, SD = 14.89) for EPAs TD1-FD6 (t{2147.52} = 0.557, P = 0.578), between faculty physicians (M = 18.3, SD = 23.71) and senior trainees (M = 15.84, SD = 14.23) for EPAs TD1-3 (t(387) = 1.236, P = 0.217), and between faculty physicians (M = 19.32, SD = 19.36) and senior trainees (M = 19.37, SD = 14.96) for EPAs FD1-6 (t{1803.37} = -0.62, P = 0.95) (Table 4).

**Table 4:** Independent t-test comparison of mean word count for positive and constructive comments written by faculty physicians and senior trainees TD: transition to discipline; FD: foundations of discipline

	Levene’s test		T-test						
	F	P-value	Faculty		Senior trainee		t	df	P-value
			Mean	SD	Mean	SD			
Positive comments									
Overall (TD1-FD6)	7.414	0.007	19.15	20.157	18.73	14.886	0.557	2147.521	0.578
TD (TD1-3)	3.192	0.075	18.3	23.711	15.84	14.225	1.236	387	0.217
FD (FD1-6)	4.729	0.03	19.32	19.361	19.37	14.96	-0.62	1803.366	0.95
Constructive comments									
Overall (TD1-FD6)	0.111	0.738	14.06	16.842	15.85	16.431	-2.528	2224	0.012
TD (TD1-3)	0.75	0.387	14.52	18.231	15.08	17.019	-0.314	387	0.754
FD (FD1-6)	0.014	0.905	13.97	16.553	16.02	16.304	-2.667	1835	0.008

When comparing overall word counts for positive comments for EPAs TD1-FD4b, ANOVA showed that there was no significant difference between faculty physicians, SMRs, and SSRs (F{2, 1906} = 1.118, P = 0.327) (Table 5). There was also no significant difference in the word count of positive comments for EPAs TD1-3, EPAs FD1-4b, and each EPA individually when comparing faculty physicians, SMRs, and SSRs (Table 5).

**Table 5:** Mean word count for positive and constructive comments written by faculty physicians, SMR, and SSR EPA: entrustable professional activity; SMR: senior medicine resident; SSR: subspecialty resident; TD: transition to discipline; FD: foundations of discipline; MS: mean squares; SS: sum of squares

Source	df	SS	MS	F	P-value
Positive comments					
TD1-FD4b					
Between groups	2	708.496	354.348	1.118	0.327
Within groups	1906	604188.179	316.993	-	-
Total	1908	604896.675	-	-	-
TD1-3					
Between groups	2	616.727	308.364	0.796	0.452
Within groups	386	149531.560	387.387	-	-
Total	388	150148.288	-	-	-
FD1-4b					
Between groups	2	634.280	317.140	1.062	0.346
Within groups	1517	452880.454	298.537	-	-
Total	1519	453514.734	-	-	-
Constructive comments					
TD1-FD4b					
Between groups	2	1588.696	794.348	2.867	0.057
Within groups	1906	528096.314	277.070	-	-
Total	1908	529685.010	-	-	-
TD1-3					
Between groups	2	373.328	186.664	0.599	0.550
Within groups	386	120206.805	311.417	-	-
Total	388	120580.134	-	-	-
FD1-4b					
Between groups	2	1899.010	949.505	3.537	0.029
Within groups	1517	407205.142	268.428	-	-
Total	1519	409104.152	-	-	-
Post-hoc analysis with Tukey HSD for constructive comments on EPAs FD1-4b					
Comparison	Mean difference	SE	P-value	95% CI low	95% CI high
SMR vs faculty	1.738	1.004	0.194	-0.62	4.09
SMR vs SSR	-0.91	1.219	0.739	-3.77	1.95
SSR vs faculty	2.648	1.076	0.037	0.12	5.17

For constructive EPA comments, comments from faculty physicians (M = 14.06, SD = 16.84) had lower word counts than senior trainees (M = 15.85, SD = 16.43) for EPAs TD1-FD6 (t{2224} = -2.528, P = 0.012). Faculty physicians (M = 13.97, SD = 16.55) also had lower word counts than senior trainees (M = 16.02, SD = 16.30) for EPAs FD1-6 (t{1835} = -2.667, P = 0.008). There was no significant difference in word counts between faculty physicians (M = 14.52, SD = 18.23) and senior trainees (M = 15.08, SD = 17.019) for EPAs TD1-3 (t{387} = -0.314, P = 0.754) (Table 4). ANOVA showed that there was a significant difference between faculty physicians, SMRs, and SSRs when comparing word counts for constructive comments for EPAs FD1-4b (F{2, 1517} = 3.537, p = 0.029) (Table 5). Post-hoc analysis with the Tukey HSD test showed that constructive comments written by SSRs (M = 16.46, SD = 14.63) had significantly higher word counts than constructive comments by faculty physicians (M = 13.81, SD = 16.46) for EPAs FD1-4b (P = 0.037). There was no significant difference (P = 0.194) between word counts for constructive comments written by SMRs (M = 15.55, SD = 17.56) and faculty physicians (M = 13.81, SD = 16.46) for EPAs FD1-4b. There was no significant difference (P = 0.736) between word counts for constructive comments written by SMRs and SSRs (M = 16.46, SD = 14.63) for EPAs FD1-4b (Table 5). ANOVA showed no significant difference in word count of constructive comments between faculty physicians, SMRs, and SSRs for EPAs TD1-FD4b, TD1-3, and each EPA individually (Table 5).

EPA comments: comparing most common phrases

For positive EPA comments, the most common two-word phrases written by faculty physicians were variations of “good job,” and the most common two-word phrases written by senior trainees were variations of “good job” and “thorough assessment.” For constructive EPA comments, the most common two-word phrases written by faculty were variations of “no concerns” and “read around,” and the most common two-word phrases written by senior trainees were variations of “no concerns” and “read around.”

## Discussion

Overall, faculty physicians gave significantly lower EPA scores compared to senior trainees, and among senior trainees, SSRs gave significantly lower EPA scores than SMRs. This relationship between faculty physicians and senior trainees was present for overall EPA scores, and remained when the TD and FD stages were considered separately. Faculty physicians gave lower scores compared to senior trainees for most individual EPAs as well.

These results support other studies in which medical students and residents gave higher scores than faculty on assessments such as OSCEs and workplace-based assessments [11-13]. For example, Hill et al. showed that faculty consultants rated medical students more strictly than specialist registrars [16]. Other studies have shown that assessors with greater seniority and rater experience have stricter scoring tendencies [17,18]. The greater seniority and rater experience of faculty physicians relative to senior trainees may explain why faculty physicians gave lower EPA scores in our study. However, conflicting literature shows that an assessor’s rater experience and trainee status do not influence such scores [19,20]. Some studies show that medical students or residents gave lower ratings than faculty physicians when evaluating their peers [14,15]. Despite these discordant studies, our study supports the idea that faculty physicians give stricter ratings than senior trainees and that this effect persists in CBME curricula and EPA scores.

The conceptual frameworks from Kogan et al. and Berendonk et al. describe multiple factors that influence how assessors make judgments and may help explain the differences in EPA scores given by faculty physicians vs senior trainees [9,10]. One major factor is the assessor’s frame of reference, which serves as the standard to which junior residents are graded against. Faculty may use their many years of clinical experience as a frame of reference when grading junior residents, with more senior faculty giving harsher ratings [8,16]. Senior trainees are still cultivating their clinical expertise as they progress through their training, and thus may grade junior residents more leniently. Another factor influencing assessors is their individual characteristics, which include academic rank and prior participation in medical education workshops. At the time of our study, the CIM program at our institution had already piloted CBD for two years, and many SMRs had themselves previously participated in CBD as junior residents. This prior experience with CBD serves as an assessor characteristic for these SMRs - they may better understand the practical challenges junior residents face when obtaining EPAs, and may be more sympathetic and lenient with assessments compared to faculty physicians.

The conceptual frameworks also describe the impact of prior relationships between assessor and learner, which alters the social context in which feedback is given and influences rating tendencies [8,9]. For example, a prior positive relationship between an assessor and learner may cause the assessor to fall victim to the “halo effect” and award higher grades. Senior trainees are in an ideal position to develop this kind of positive relationship with junior residents, as they are more accessible to junior residents compared to faculty physicians and are closer to junior residents in training [21-25]. Additionally, senior trainees may be reluctant to provide negative feedback for fear of impairing social relationships with their junior residents [9,26]. The development of such close working relationships can influence senior trainees to give more lenient assessments compared to faculty.

Overall, senior trainees had higher word counts for constructive comments for EPAs compared to faculty physicians. These results are similar to those found by Ringdahl et al, where senior residents were more likely than senior faculty members to write negative comments when evaluating PGY1 residents [27]. Even though this difference between senior trainees and faculty physicians in the word counts of constructive comments is statistically significant, a difference of two words per comment is unlikely to improve the quality of feedback. This is supported by the fact that the most common phrases for both positive and constructive comments were similar between faculty and senior trainees, suggesting little difference in feedback content.

This study does have limitations. We only reviewed EPAs observed for CIM PGY1 residents, and the difference between faculty physicians and senior trainees may not be as prominent in other disciplines. EPA data was only collected from a single institution, and only over one year. We also gathered data from the first year of CBD implementation in CIM programs in Canada. As faculty physicians and senior trainees become more accustomed to the tasks involved with CBD, the differences in EPA scores and comments may change. This study also does not address how important this difference is. While perhaps implied that staff feedback is more accurate, this is also unclear from this study. By allowing senior residents to assess junior residents, do the higher scores assigned truly reflect their performance better or worse than staff assessors? If the scores are more lenient, further study may be critical to determine the impact of these results. Does this mean more junior residents are promoted in the CBD framework without meeting competency? Does this mean junior residents may seek assessment from their senior residents more often than staff assessors? Is further assessor education needed to normalize assessment? While this study does not answer these questions, the results open the opportunity for further review.

## Conclusions

Compared to senior trainees, faculty physicians gave significantly lower EPA scores and wrote significantly shorter constructive comments with their EPAs. The next steps for future research include expanding the number of residents involved to include multiple programs and disciplines. Residents from multiple sites should also be studied to examine for more generalizable results. Deeper analysis to determine if other factors impart a role in these results is also important, including the potential role of assessor and trainee gender, age, teaching, and clinical experience, as well as the role of EPA burden. If similar results are identified, educational leaders will need to consider the impact it has on the ongoing rollout of competency-based medical education to ensure residents are being assessed and provided feedback as intended.
